# Atrophie cérébrale diffuse au cours d'un syndrome de Goldenhar: à propos d'un cas

**DOI:** 10.11604/pamj.2014.19.139.5231

**Published:** 2014-10-13

**Authors:** Yogolelo Asani, Cilundika Mulenga, Léon Kabamba Ngombe, Mbuyi-Musansayi, Kalenga Muenze, Chenge Borasisi, Speeg-Schatz

**Affiliations:** 1 Université de Lubumbashi, Faculté de Médecine, Département des Spécialités, Service d'Ophtalmologie, RD Congo; 2 Université de Lubumbashi, Faculté de Médecine, Département de Santé Publique, RD Congo; 3 Université de Kamina, Faculté de Médecine, Département de Santé Publique, Unité de Toxicologie, RD Congo; 4 Université de Lubumbashi, Faculté de Médecine, Département de Chirurgie, RD Congo; 5 Université de Lubumbashi, Faculté de Médecine, Département de Gynécologie et Obstétrique, RD Congo; 6 Université de Strasbourg, Faculté de Médecine, Service d'Ophtalmologie, France

**Keywords:** Syndrome de Goldenhar, atrophie cérébrale diffuse, nourrisson, Goldenhar syndrome, diffuse brain atrophy, infant

## Abstract

Les auteurs rapportent un cas d'un syndrome de Goldenhar non décris dans la littérature chez un nourrisson de 3 mois, de sexe féminin présentant une atrophie cérébrale diffuse. Cette observation permet de décrire cette malformation rare et d'attirer l'attention de l'opinion scientifique à mettre en marche des études poussées afin de comprendre la survenue de cette pathologie.

## Introduction

Le syndrome de Goldenhar ou dysplasie oculo-auriculo-vertébralea été découvert par Maurice Goldenhar en 1952. Il s'agit d'une maladie rare, sporadique avec une étiologieidiopathique [1]. C'est un syndrome malformatif en rapport avec des anomalies de développement du premier arc branchial [2]. Dans notre milieu,il n'y a pas des données relatives à cette malformation. Le but de ce travail est de décrire cette malformation, et d′attirer l′attention de l′opinion scientifique à mettre en marche des études poussées afin de comprendre la survenue de cette pathologie.

## Patient et observation

**Figure 1:**
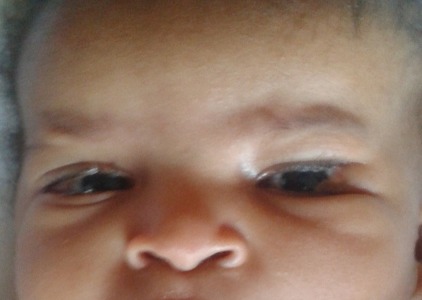
Un dermoîde du limbe de 10-11h à l’œil droit

**Figure 2:**
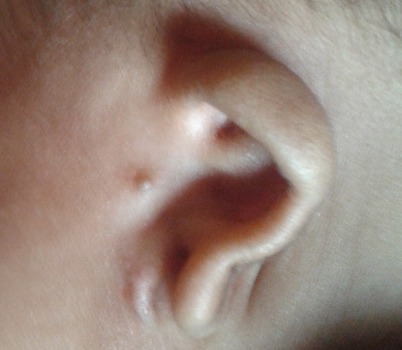
Appendice pré auriculaire gauche ainsi qu'une déformation du pavillon de l'oreille gauche

**Figure 3:**
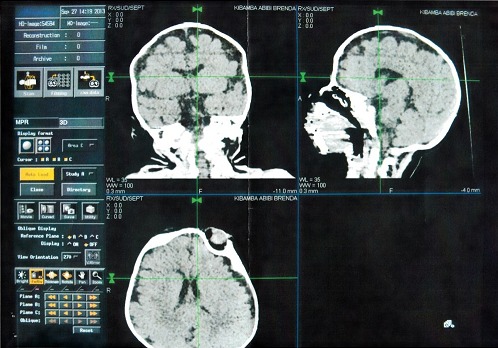
Atrophie cérébrale

Nous avons consulté un nourrisson âgé de 3 mois amené par sa mère en consultation, qui a présenté comme plaintes sécrétion et larmoiement des yeux depuis sa naissance. A notre examen physique, nous avons objectivé un dermoîde du limbe de 10-11h à l'oeil droit (Figure 1), un colobome de paupière à l'oeil gauche avec appendice pré auriculaire gauche ainsi qu'une déformation du pavillon de l'oreille gauche par rapport à l'oreille droite (Figure 2); un épicanthus avec aplatissement de la base du nez. La radiographie dorsolombaire a révélé une malformation de la colonne vertébrale consistant en une hémi-vertèbre au niveau dorsal et L4- L5. Le CT Scan cranio-cérébral a trouvé une atrophie cérébrale diffuse (Figure 3).

## Discussion

Le syndrome de Goldenhar survient le plus souvent de façon sporadique [3]. Il est associé à des altérations osseuses et surtout des parties molles. Il existe de fois une dysmorphie faciale avec macrostomie, micrognathie, des anomalies auriculaires (duplication du tragus, appendice pré auriculaire, fistule auriculaire). D'autres atteintes sont plus rares (spina bifida, retard mental: 10%). Certains auteurs ont signalé qu'en dehors du classique syndrome de Goldenhar (atteinte des yeux, oreilles et des vertèbres), on note la présence des certaines anomalies notamment: rénales, gastro-intestinales, d'une fente labiale et palatine, d'une malformation de l'articulation temporale et la malocclusion buccale [4]. Par contre Healey et al. ont observé des anomalies de la colonne cervicale avec un taux élevé de l'instabilité de C1-C2, la présence des hémi-vertèbres et les échecs de la segmentation qui étaient beaucoup plus fréquents et aboutissaient à une scoliose thoracique conduisant à la fusion spinale [5]. Ce qui n'est pas le cas avec notre observation.

Cependant, Schrander Stumpel et al.ont signalé la présence de l'hydrocéphalie associée à la triade oculo-auriculo-vertébrale tout en constatant d'autres anomalies (cardiaques, fentes labiales, fentes palatines, anophtalmie, microphtalmie, retard mental) [6]. Selon la littérature,l'atteinte ophtalmologique est dominée par les colobomes palpébraux et les dermoîdes du limbe [3]. Cependant, d'autres atteintes peuvent être rencontrées: ptosis, obliquité anti-mongoloïdes des paupières. Par ailleurs, la présence de l'atrophie cérébrale diffuse non classique de ce syndrome dans la littérature scientifique de notre pays, attire notre attention. D'une manière générale, le traitement des lésions oculaires est chirurgical. Ainsi, les résultats dépendent de la sévérité de la dysmorphie faciale [3].

## Conclusion

Le syndrome de Goldenhar est l'un des syndromes le moins observé en pratique courante dans notre milieu. En effet, l'atteinte ophtalmologique est dominée par le colobome palpébral et les dermoîdes du limbe. L'atteinte extra oculaire est dominée par les anomalies auriculaires, dont la plus constante est l'appendice pré-tragien. L'atrophie cérébrale diffuse a été observée dans notre cas. Le rôle de l'ophtalmologique est important dans le diagnostic positif.
